# Tissue-specific transcriptomics, chromosomal localization, and phylogeny of chemosensory and odorant binding proteins from the red flour beetle *Tribolium castaneum* reveal subgroup specificities for olfaction or more general functions

**DOI:** 10.1186/1471-2164-15-1141

**Published:** 2014-12-18

**Authors:** Stefan Dippel, Georg Oberhofer, Jörg Kahnt, Lizzy Gerischer, Lennart Opitz, Joachim Schachtner, Mario Stanke, Stefan Schütz, Ernst A Wimmer, Sergio Angeli

**Affiliations:** Department of Developmental Biology, Georg-August-University Goettingen, Johann-Friedrich-Blumenbach-Institute for Zoology and Anthropology, GZMB, Ernst-Caspari-Haus, Justus-von-Liebig-Weg 11, Goettingen, 37077 Germany; Department of Forest Zoology and Forest Conservation, Buesgen-Institute, Georg-August-University Goettingen, Buesgenweg 3, Goettingen, 37077 Germany; MPI for Terrestrial Microbiology, Karl-von-Frisch-Straße 10, Marburg, D-35043 Germany; Institute for Mathematics and Computer Science, University of Greifswald, Walther-Rathenau-Straße 47, Greifswald, D-17487 Germany; Functional Genomics Center Zurich, Winterthurerstr. 190, Zurich, 8057 Switzerland; Department of Biology - Animal Physiology, Philipps-University Marburg, Karl-von-Frisch-Str. 8, Marburg, 35032 Germany; Faculty of Science and Technology, Free University of Bolzano, Piazza Università 5, Bolzano, 39100 Italy

**Keywords:** Chemosensory protein (CSP), Gustation, Odorant binding protein (OBP), Olfaction, Proteome, Transcriptome, *Tribolium castaneum*

## Abstract

**Background:**

Chemoreception is based on the senses of smell and taste that are crucial for animals to find new food sources, shelter, and mates. The initial step in olfaction involves the translocation of odorants from the periphery through the aqueous lymph of the olfactory sensilla to the odorant receptors most likely by chemosensory proteins (CSPs) or odorant binding proteins (OBPs).

**Results:**

To better understand the roles of CSPs and OBPs in a coleopteran pest species, the red flour beetle *Tribolium castaneum* (Coleoptera, Tenebrionidae), we performed transcriptome analyses of male and female antennae, heads, mouthparts, legs, and bodies, which revealed that all 20 CSPs and 49 of the 50 previously annotated OBPs are transcribed. Only six of the 20 CSP are significantly transcriptionally enriched in the main chemosensory tissues (antenna and/or mouthparts), whereas of the OBPs all eight members of the antenna binding proteins II (ABPII) subgroup, 18 of the 20 classic OBP subgroup, the C + OBP, and only five of the 21 C-OBPs show increased chemosensory tissue expression. By MALDI-TOF-TOF MS protein fingerprinting, we confirmed three CSPs, four ABPIIs, three classic OBPs, and four C-OBPs in the antennae.

**Conclusions:**

Most of the classic OBPs and all ABPIIs are likely involved in chemoreception. A few are also present in other tissues such as odoriferous glands and testes and may be involved in release or transfer of chemical signals. The majority of the CSPs as well as the C-OBPs are not enriched in antennae or mouthparts, suggesting a more general role in the transport of hydrophobic molecules.

**Electronic supplementary material:**

The online version of this article (doi:10.1186/1471-2164-15-1141) contains supplementary material, which is available to authorized users.

## Background

The red flour beetle *Tribolium castaneum* (Herbst, Coleoptera, Tenebrionidae) is a secondary pest of stored, dried food products [1]. As a coleopteran model system, it represents the largest insect order, containing many different pests like bark beetles (*Dendroctonus ponderosae, Ips typographus*), colorado potato beetle (*Leptinotarsa decemlineata*), pollen beetle (*Brassicogethes aeneus*) and the Western corn rootworm (*Diabrotica virgifera*), which cause severe economic and ecological damage. Over the past years, *T. castaneum* turned into a remarkable model organism with plenty of genetic tools such as systemic RNA interference [2, 3], forward genetics based on insertional mutagenesis [4], transgene-based mis-expression systems [5, 6], as well as a fully annotated genome sequence [7, 8]. These tools predestine *T. castaneum* as a model system for coleopterans and to investigate findings from the vinegar fly *Drosophila melanogaster* for their generality in insects.

Odor discrimination is a key process in insect life: from food and host finding to partner recognition, insects rely strongly on odor stimuli. Perception of odorants takes place in the chemosensory (olfactory or gustatory) sensilla and is supposed to be mediated by chemosensory proteins (CSPs) or odorant binding proteins (OBPs) [9–13], followed by detection via odorant receptors (ORs), ionotropic glutamate-like receptors (IRs), or gustatory receptors (GRs) [14]. The olfactory sensilla are hair like structures with the highest density on the antennae. They are housing the dendrites of the odorant receptor neurons and are filled with aqueous lymph. This lymph is secreted by non-neuronal auxiliary cells and contains some CSPs and OBPs [15, 16]. The CSPs and OBPs are small (10 to 30 kDa), globular, and water soluble proteins [17] providing a hydrophobic pocket for ligand binding [18]. The CSPs are characterized by four, conserved cysteine residues forming two disulfide bonds (C_1_-C_2_, C_3_–C_4_) [19]. The OBPs – classic OBPs and antennal binding proteins (ABPIIs) [17] – have six highly conserved cysteine residues forming three interlocking disulfide bonds (C_1_–C_3_, C_2_–C_5_, C_4_–C_6_) between six α-helices, conferring a high stability to these proteins [18, 20]. The C-OBPs seem to be derived from classic OBPs and are lacking the C_2_–C_5_ disulfide bridge [17, 21–23].

It is believed that hydrophobic semiochemicals interact first with CSPs or OBPs to get shuttled through the aqueous sensillar lymph and to finally reach and activate ORs [14]. Besides evidence that CSPs are involved in chemoreception of the alfalfa plant bug *Adelphocoris lineolatus* and the Japanese carpenter ant *Camponotus japonicus* [24, 25] and their presence in the antennae of various species [10, 13, 26–29], there are no functional experiments conducting a role in chemo-sensation. In contrast, the involvement of OBPs in olfaction has been verified by several functional studies: experiments conducted with moth pheromone receptors in heterologous expression systems [30–32] or *in vivo* using the *Drosophila melanogaster* “empty neuron system” [33, 34] revealed that the presence of the corresponding OBP (pheromone binding protein, PBP) increases the sensitivity to the pheromone by 2 to 3 orders of magnitude (reviewed in [14]). Additionally, *D. melanogaster* mutants for the OBP Lush [35], allelic variation of different OBPs in *D. melanogaster* [36] and of an OBP in the fire ant *Solenopsis invicta* [37], as well as several RNAi based experiments in *D. melanogaster* and mosquitoes [38–40] showed that OBPs are important for the correct and highly sensitive reception of different semiochemicals in these insects, but might not be absolutely essential [41]. However, expression analysis of different insects have revealed that CSPs and OBPs are not restricted to the main chemosensory tissues [42–48] but are also involved in other tasks, e.g. the release of semiochemicals [49], mating [50], embryogenesis [51], immune-response [52], and regeneration [53]. Moreover, olfactory based systems [54] such as OBP coupled biosensors might improve pest and plant disease monitoring [55, 56], risk assessment [57], or prevent infestation by camouflaging or repelling [58, 59]. This could offer novel eco-friendly and cost effective ways to combat the fast adaption of *Tribolium* against several insecticides and respective resistance development [60, 61] and thus improve the protection of stored agricultural products [62] against migrating beetles [63].

In this study we use tissue-specific transcriptomics to improve the genome annotation of the *T. castaneum* CSPs and OBPs and to determine their expression profile. We place these data into a phylogenetic context in order to get better insights into their potential functions with a comparative evolutionary perspective.

## Methods

### *Tribolium*rearing

*T. castaneum* strain San Bernardino (Herbst, 1797; Insecta, Coleoptera, Tenebrionidae), was reared on organic wheat flour supplemented with 5% yeast powder at 28°C and 40% relative humidity under constant light. The Beetles were collected from different breeding boxes varying in age (up to three month) and culture density.

### RNA isolation and sequencing

From the sex separated and age pooled animals about 1000 antennae, 600 legs, 150 mouthparts (as piece of the head capsule anterior of the antennae), 50 heads (the whole head capsule excluding the antennae) and 20 bodies (excluding head and legs) were manually dissected and immediately transferred to ice cold RNA lysis buffer (Zymo Research, Irvine, USA). For larval tissues about 100 heads and 50 bodies of unsexed last instar larvae were collected. Total RNA was isolated using the ZR Tissue & Insect RNA Micro Prep Kit (Zymo Research, Freiburg, Germany) following the manufacturer’s protocol. The Library preparation for RNA-Seq was performed using the TruSeq RNA Sample Preparation Kit (Illumina, San Diego, USA) starting from 300 ng of total RNA. Accurate quantification of cDNA libraries was performed by using the QuantiFluor™ dsDNA System (Promega, Fitchburg, USA). The size range of final cDNA libraries (280 bp) was determined applying the DNA 1000 chip on the Bioanalyzer 2100 (Agilent, Santa Clara, USA). cDNA libraries were amplified and sequenced using the cBot and HiSeq2000 from Illumina (paired end; 2×100 bp). Sequence images were transformed with Illumina software BaseCaller to bcl files, which were demultiplexed to fastq files with CASAVA v1.8.2 (Illumina). Quality check was done via fastqc (v. 0.10.0, Babraham Institute, Cambridge, UK).

### OBP and CSP re-annotation, SNP calling and mapping

The obtained fastq formatted Illumina reads were mapped to the *Tribolium castaneum* 3.0 official gene set using bowtie2 [64] with the “very-sensitive” presetting. The previously published CSP and OBP sequences [7, 17, 21, 65] were identified in this gene set with blastp [66] implemeted in bioperl [67]. Samtools mpileup (v0.1.18) [68] was used to check the RNAseq data for SNPs and indels. In a genome independent approach a de novo assembly was built. Quality filtering was performed with the NGSQC Toolkit (v2.3.1) [69] in three steps: 1) removal of reads containing ambiguous bases with AmbiguityFiltering.pl, all settings default; 2) trimming of bad quality bases from 3′ ends with TrimmingReads.pl, -q 28 -n 60; 3) removal of bad quality reads with IlluQC_PRLL.pl, N 5 -l 90 -s 24. Before the assembly the reads were digitally normalized using the normalize_by_kmer_coverage.pl script from trinity (release2013_08_14) [70] with these settings: --max_cov 50 --pairs_together. The assembly was performed with Trinity.pl, all settings default. Translations of open reading frames were extracted with transcripts_to_best_scoring_ORFs.pl. The preliminary re-annotation of the whole *T. castaneum* gene set (au3) was generated by the gene finder AUGUSTUS [71]. Alignments of RNA-Seq reads from libraries from several tissues, stages and conditions (e.g. embryo, larva head, larva body, early and late stage pupa, adult antenna, leg, head, body, stink glands, ovary) were incorporated. These data were produced mainly by the iBeetle consortium [72] and a separate publication is in preparation describing the re-annotation of the whole gene set of *T. castaneum* based on these RNA-Seq data, which can be viewed in a respective genome browser [73]. It contains a track with the au3 gene models as well as RNA-Seq coverage tracks of different stages including the data collected for this study. In total 1,624,983,955 reads were mapped against the genome with the alignment tool BLAT [74]. The read alignments were filtered, so that only alignments of reads that mapped uniquely to the genome and that showed a percent identity of at least 93% were kept. Paired reads were required to be aligned in the correct orientation and with a maximal genomic distance of 500,000 base pairs. Intron evidence was collected based on reads with a spliced alignment against the genome and evidence for transcription is taken from RNA-Seq covered regions. In an iterative process, the SNP calling, the de novo assembly, and the au2 gene set were used to manually curate the OBP and CSP sequences based on previously published annotations [7, 17, 21, 65]. The corresponding au3 gene models were replaced with these new candidate sequences and the resulting modified au3 gene set was used to remap the RNAseq data with bowtie2 using the ‘very-sensitive’ presetting. Finally all sequences were searched for signal peptides using the SignalP4.1 server [75] and browsed for conserved functional domains [76].

### *Tribolium castaneum*expression profiling

The mapped reads of the re-annotated OBPs and CSPs in the particular tissue or sex sample were counted with samtools [68]. To normalize the count numbers RPKM values were calculated and plotted as log2 [RPKM + 1] (Additional file 1: Table S1). The values were visualized using the matrix2png interface (version 1.2.1; [77]) and the figures were composed with inkscape [78]. Male and female reads from the sequenced tissues were pooled and considered as biological replicates. Statistical analysis of the data was performed in R [79] using the DESeq package (version1.12.0) [80] from bioconductor [81]. All sequenced tissues were compared to body as reference. Significant differentially expressed genes (false discovery rate < 0.05) are marked with asterisks. For the intersex comparison the two male and three female replicates of antenna were treated the same way.

### Phylogenetic analysis and interspecies comparison

We compared our sequences on protein level with data from *D. melanogaster* and the malaria mosquito *Anopheles gambiae* obtained from Vieira and Rozas 2011 [17]. After subtraction of the signal peptide (SignalP4.1) [75], the sequences were aligned using MAFFT v7.040b [82] as described [17] and the tree was constructed using RAxML version 7.8.6. [83] with the LG substitution model in the case of the CSPs or the VT substitution model for the OBPs and GAMMA correction. Node support was assessed with 100 rapid bootstrap replicates. The relative expression levels were calculated as log2 fold changes of antenna/body and palp (mouthpart)/body. For *T. castaneum*, log2FC data from inner species comparison were used. The *D. melanogaster* data set was downloaded from EMBL gene expression atlas [84] originally published in Farhadian et al. 2012 [43] and the *An. gambiae* data were obtained from Pitts et al. 2011 [42]. The phylogenetic tree was visualized by iTOL [85] and descriptions were added using inkscape [78]. Since the absolute expression levels of the different candidates are lost in the depiction of the fold changes, we provide them in Additional file 2: Figure S1. Please note that the methods used to obtain the different expression data (RNA-seq and microarray) are not directly comparable. Therefore, Additional file 2: Figure S1 can just give an impression on more or less abundant transcripts.

### Cloning of selected OBPs and CSPs open reading frames

Manually separated heads were ground in liquid nitrogen, and total RNA was extracted using the TRIZOL reagent (life technologies, Carlsbad, USA). Messenger RNA was purified with the Dynabeads purification kit (life technologies, Carlsbad, USA) and cDNA was synthesized using the Super-Script first-strand synthesis system (life technologies, Carlsbad, USA). Hotstart Taq DNA polymerase (Qiagen, Venlo, Netherlands) was used to amplify individual transcripts. Finally the products were cloned into PCR2.1 vector (life technologies, Carlsbad, USA) and verified by sequencing. Most primers were designed to bind within the UTRs to not bias start and stop codons and are summarized in Additional file 1: Table S1.

### MALDI-TOF MS

For identification of OBPs and CSPs on protein level, about 400 antennae per sample were manually separated and homogenized in 200 μl milliQ water containing 0.1% trifluoroacetic acid (Sigma-Aldrich, St. Louis, USA) with a tube fitting pestle. To get rid of the debris, the samples were centrifuged and 150 μl supernatant was used further. To break down the secondary structure, the disulfide bridges were reduced and simultaneously the cysteine-derived thiol groups alkylated with 10 μl 100 mM tris (2-carboxyethyl) phosphine hydrochloride (Chemos GmbH, Regenstauf, Germany), 10 μl 200 mM 2-vinylpyridine (in 30% acetonitrile, Sigma-Aldrich) and 26 μl 8 M guanidine hydrochloride (Sigma-Aldrich) for 8 min at 35°C, followed by additional incubation for 30 min. at pH 8 after adding 11 μl 1 M ammonium bicarbonate (Sigma-Aldrich). The sample was loaded on a VIVASPIN 500 VS011 Ultrafiltration unit (5000 MWCO, Sartorius, Goettingen, Germany) and centrifuged for 10 min followed by two washing steps with 200 μl milliQ. For storage at -20°C over night the remaining 50 μl sample was mixed with 100 μl milliQ and 50 μl acetonitrile. After 30 min of centrifugation, 100 μl milliQ, 50 μl 50 mM Ammonium bicarbonate and 20 μl acetonitrile were added and debris was removed by additional centrifugation. Digestion took place in the remaining volume over the membrane by adding 0.11 μg sequencing-grade modified trypsin (Promega, Fitchburg, USA) and the resulting peptides were eluted. The mixture was analyzed by nanoLC (PepMap100 C-18 RP nanocolumn and UltiMate 3000 liquid chromatography system; Dionex, Sunnyvale, USA) and automated MSMS (4800 Proteomics Analyzer MDS¸ AB Sciex, Framingham, USA). MSMS data were searched against the au2 gene set (http://bioinf.uni-greifswald.de/gb2/gbrowse/tcas4/) using Mascot embedded into GPS explorer software (AB Sciex). Identified proteins, their scores, and Pfam predictions are provided in Additional file 3: Table S2.

## Results and discussion

### Re-annotation and re-naming of *Tribolium*CSPs and OBPs

**Figure 1:**
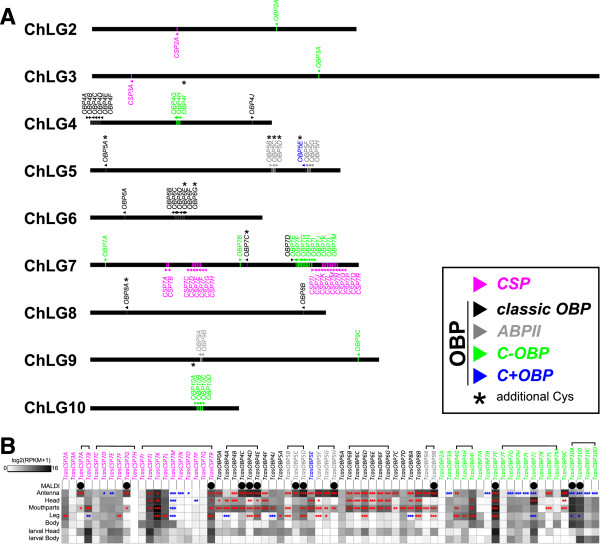
**Chromosomal localization and chemosensory expression profile of** ***Tribolium*** **CSPs and OBPs. (A)** Chromosomal localization of CSPs (magenta), classic OBPs (black), ABPII (grey), C-OBPs (green) and C + OBP (blue), based on Georgia GA-2 strain genome assembly 3.0 [7]. The arrowheads indicate the orientation of the genes from 5′►3′. Genes encoding for more than the six highly conserved cysteines (or four in case of C-OBP) are labeled with an asterisk (*). (**B)** Heatmap showing the absolute expression level of the OBPs/CSPs as log2 (RPKM + 1) in different tissues (adult antennae, head (missing antennae but including mouth parts), mouthparts, legs, body, as well as larval head and body). The candidates are blotted according to their chromosomal localization, horizontal brackets above indicate clustering in the genome. A black dot in the first row labeled ‘MALDI’ indicates that at least one unique tryptic fragment of the particular candidate was identified in an antennae sample on protein level. The expression levels are represented by a log2 greyscale with high expression levels (2^16^ RPKM) labelled black. The asterisks mark statistically significant differtially expressed genes compared to body. The red asterisks represent up- and the blue down-regulation (p-values are * < 0.05; ** < 0.01; *** < 0.001).

In the past, several authors published sequences of *Tribolium* CSPs and OBPs based on computational predictions [7, 17, 21, 65] resulting in different conflicting annotations and designation. We revised the originally described 20 CSPs and 50 OBPs using transcriptome analysis of different tissues including antennae and mouthparts. Subsequently we applied a new nomenclature to prevent confusion and to provide a unique and distinguishable nomenclature following the one used for *Drosophila* OBPs [22]. We used the prefaces CSP, and OBP to reflect the fact that a gene is a member of one of these protein families. This is followed by a number reflecting the chromosomal location and a letter that conveys its relative position on the chromosome (Figure 1A). Thus, the new name OBP9B refers to the second OBP on the ninth chromosome. A comparative list putting all previous names in relation can be found in the Additional file 1: Table S1.

We detected reads corresponding to all previously described *Tribolium* CSPs and OBPs except TcOBP2A. Accordingly we confirmed or corrected the predicted open reading frames of all 20 CSPs and 49 OBPs. In case of low abundant transcripts with poor read coverage in our samples we used additional data obtained from embryo and pupa to support the re-annotation (iBeetle genome browser) [73]. The comparison of the latest genome based annotation [17] with our transcriptome based re-annotation revealed differences mainly based on wrongly predicted intron-exon boundaries. The identified discrepancies to previous annotations in the OBPs and one CSP did not cause severe differences in the phylogenetic relationship. However, they partially affect in addition to the intron-exon boundaries also the start or stop codons, which could impair cloning efforts for further investigations. In addition, wrong indels can cause differences in three-dimensional modeling of OBPs and by this also affect predictions in respect to potential ligands. Therefore, we point out clearly which annotations of previously identified OBPs and CSPs needed to be changed: Due to an unpredicted intron in the 5′UTRs of TcCSP2A, TcOBP0A and TcOBP5F the start codons had to be changed. In case of TcOBP5E, TcOBP6A, TcOBP6E, TcOBP6F, TcOBP8A, TcOBP7G and TcOBP7H wrong or un-predicted introns led to insertions or deletions not affecting the highly conserved cysteine pattern. In TcOBP4A, TcOBP4C, TcOBP4E, TcOBP4F, TcOBP5C, TcOBP5D and TcOBP5H an incorrect last intron caused differences in the C-terminal region, that was supposed to be involved in ligand binding and release [86, 87]. Additionally we were able to revive previously wrongly annotated pseudogenes. Formerly termed OBP49P is an intact gene with some characteristics of an OBP and is now called TcOBP7C. OBP50P [17] turned out to correspond to the already described OBP-C08 [7] and is now called TcOBP7K. The originally termed CSP21P, which is derived from a duplication of the first exon of TcCSP7G, is expressed at low levels and now called TcCSP7H, which either represents an expressed pseudogene as observed in *Nasonia* [88] or is a truncated CSP. Because of its high sequence similarity to TcCSP7G we were only able to map unique reads to the 3′UTR. Therefore, expression of TcCSP7H is not indicated in our figures. To confirm the new annotation we checked the resulting protein sequences for common characteristics of CSP- or OBP-like signal peptide, conserved domains, size and cysteine composition [89] and we cloned the whole open reading frame of 33 of the 70 candidates from cDNA. All newly annotated proteins contain a predicted N terminal signalpeptide with an length of 15 to 22 AA (SignalP 4.1 [75]). According to the conserved domain database [76], all re-annotated CSPs and OBPs (except the C + OBP, OBP5E) are members of the OS-D respectively PBP_GOBP superfamily (Additional file 1: Table S1).

The re-annotated CSPs of *Tribolium* have an average size of 128 AA (110 AA mature CSP), they range from the smallest, TcCSP7C, with just 99 AA to the longest, TcCSP7E, with 251 AA. On average, *Tribolium* CSPs are within the size range of other species [12]. The cysteine formula of the *Tribolium* CSPs follows the highly conserved pattern with four cysteines arranged by an exact spacing of C1X_6_C2X_18_C3X_2_C4 [17]. The only exception is the pseudo/truncated gene TcCSP7H that stops after C2.

The OBPs are slightly longer and vary from 106 up to 320 AA with an average size of 143 AA (125 for the mature OBP), which is similar to other insects such as *D. melanogaster* (117 up to 245 AA) or *An. gambiae* (107 up to 356 AA [17]). Most of the *T. castaneum* classical OBPs (including ABPIIs) show a conserved cysteine pattern (C1X_24-29_C2X_3_C3X_22-43_C4X_8-10_C5X_8_C6) comparable to *D. melanogaster* [22] and other insects. The only exceptions are the C + OBP (TcOBP5E, 241 AA) and the non-clustered OBPs TcOBP5A, TcOBP7C, and TcOBP8B (Figure 1A), which differ in C spacing, are of unusual length (TcOBP5E, 241AA; TcOBP5A, 176 AA; TcOBP7C 320 AA; TcOBP8B, 200 AA), and are phylogenetically close to C + OBPs. The one typical C + OBP, TcOBP5E, has an expanded N and C terminus containing six additional cysteines, whereas TcOBP7C has an expanded N terminus, TcOBP8B extra AAs between C1 and C2, TcOBP5A between C1 and C2 plus between C4 and C5. TcOBP5A, TcOBP8B, and TcOBP5E have extra cysteines 17–19 AA before C1 and nine AA after C6. The OBPs TcOBP5B, TcOBP5C, TcOBP5D, TcOBP6E, TcOBP6G, TcOBP7C, and TcOBP8A contain also at least one additional cysteine. TcOBP6G and the ABPIIs (TcOBP5B, TcOBP5C, TcOBP5D) have a conserved additional cysteine seven AA after C3. Despite the increased amount of cysteines only TcOBP5E carries the typical proline residue following C6 (C6b) [90, 91]. The C-OBPs show a conserved cysteine pattern, with only four cysteines, lacking C2 and C5 (C1X_18-30_C3X_37-39_C4X_16-17_C6). We can conclude that all re-annotated CSPs (except CSP7H) and OBPs fulfill the rigid criteria previously defined based on other species [22, 89].

### Expression profile of the CSPs and OBPs in *Tribolium castaneum*

As several CSPs and OBPs are supposed to be involved in olfaction, we comparatively analyzed the expression of these genes in the main chemosensory tissues antennae and mouthparts (here defined as the piece of the head capsule anterior to the antennae) plus in heads (the whole head capsule excluding the antennae), legs, and bodies (excluding head and legs) of males or females, respectively. To get some first insights into expression differences between larval and adult stages, we also sequenced heads including antennae and bodies (without head) of last instar larvae. The results as log2 RPKM are represented as heat-map in Figure 1B.

The expression of the majority of the CSPs is detected in a wide variety of tissues. Transcripts of only five of the 20 CSPs are significantly enriched in antennae (*Tc*CSP7A, *Tc*CSP7G, *Tc*CSP7J, *Tc*CSP7K, and *Tc*CSP7R) and six in the mouthparts (*Tc*CSP7A, *Tc*CSP7B, *Tc*CSP7G, *Tc*CSP7J, *Tc*CSP7K, and *Tc*CSP7R). However, only the expression of *Tc*CSP7A and *Tc*CSP7G is restricted to the main olfactory tissue. *Tc*CSP7I and *Tc*CSP7M are exclusively expressed in the body. Six of the CSPs showed no or only poor expression in our tissue samples, however, we found them expressed at other developmental stages by searching the iBeetle genome browser [73]. *Tc*CSP7P and *Tc*CSP7Q are expressed in embryo and pupa, *Tc*CSP3A mainly in embryo, *Tc*CSP7N in embryo and larva, and *Tc*CSP7O only in larva.

In contrast to the CSPs, the expression of the OBPs is more restricted to the main chemosensory tissues (antennae and mouthparts). All eight ABPIIs are highly expressed in the antennae. With the exception of *Tc*OBP5C, all of them are also significantly enriched in mouthparts indicating an involvement of this subgroup in chemo-reception (Figure 1B). 15 of the 20 classic OBPs are significantly enriched in antennae and mouthparts (Figure 1B, *Tc*OBP0A, *Tc*OBP4B, *Tc*OBP4C, *Tc*OBP4D, *Tc*OBP4E, *Tc*OBP4F, *Tc*OBP6B, *Tc*OBP6C, *Tc*OBP6D, *Tc*OBP6E, *Tc*OBP6F, *Tc*OBP6G, *Tc*OBP7C, *Tc*OBP8A, *Tc*OBP8B), whereas three are enriched only in the mouthparts (*Tc*OBP4A, *Tc*OBP4J, *Tc*OBP7D). Only three of the classic OBPs are evenly expressed in all tissues. Interestingly, these are the non-clustered ones (*Tc*OBP5A, *Tc*OBP6A, *Tc*OBP7D). The C + OBP (*Tc*OBP5E) is enriched in mouthparts and in antennae. Most of the 21 C-OBPs are expressed in all tissues similar to the majority of the CSPs, only five are significantly enriched in antennae and mouthparts compared to body with three of them also highly abundant in head or leg (*Tc*OBP4G, *Tc*OBP4I, *Tc*OBP7E). Thus, there are just two C-OBPs (*Tc*OBP7L and *TcOBP9C*) most likely exclusively involved in chemosensory processing. Ten C-OBPs are significantly down-regulated in the antennae compared to the body: *Tc*OBP3A, *Tc*OBP7B, *Tc*OBP7G, *Tc*OBP7H, *Tc*OBP7I, *Tc*OBP7J, *Tc*OBP10A, *Tc*OBP10B, *Tc*OBP10C, and *Tc*OBP10D. *Tc*OBP2A expression was not detected at all, *Tc*OBP7F is expressed during metamorphosis and *Tc*OBP7B is mainly active during embryogenesis as well as metamorphosis.

**Figure 2:**
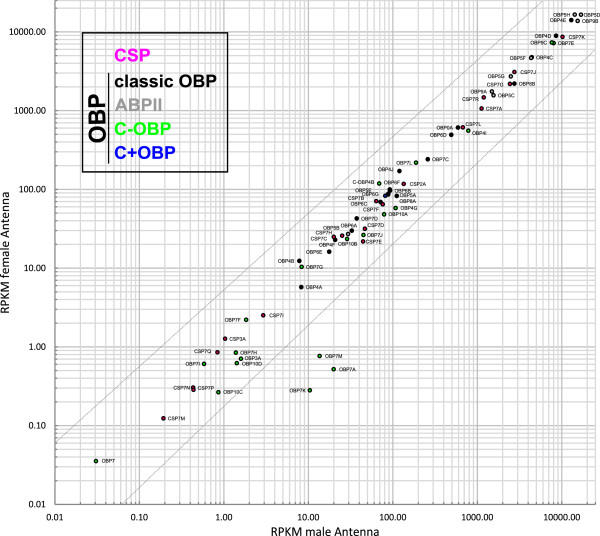
**Comparison of expression levels of CSPs and OBPs in male and female antennae, average values based on two male and three female antennal samples.** Scatter plot of the RPKM values of the CSPs (in pink) and OBPs (classic in black, ABPII in grey, C-OBP in green, C + OBP in blue). The dotted lines represent a fivefold difference.

Statistical analysis of the two male and three female antennal samples did not show any significant difference, due to the low abundance of potential candidates and the relative high dispersion of the samples (Figure 2; Additional file 4: Figure S2, Additional file 5: Figure S3, Additional file 6: Figure S4 and Additional file 7: Figure S5). Nevertheless*, Tc*OBP7A, *Tc*OBP7K, and *Tc*OBP7M were more than five-fold overexpressed in male antennae and could be interesting for further investigation (Figure 2). The fact that we found no major and significant differences between male and female is consistent with anatomical data from the antennal lobe were no sexual dimorphism was found [92], and the antennal morphology from a related species, *Tribolium brevicornis*, were both sexes are anatomically similar [93].

The comparison of transcriptome data of adult antennae and larval heads revealed differences in the expression of five ABPIIs (*Tc*OBP5C, *Tc*OBP5F, *Tc*OBP5G, *Tc*OBP5H, and *Tc*OBP9A), one classic OBP (*Tc*OBP4D) and five C-OBPs (*Tc*OBP3A, *Tc*OBP4G, *Tc*OBP4I, *Tc*OBP7I, and *Tc*OBP9C). Most of these transcripts were present in adult antennae or mouthparts but were absent in the larval head (Figure 1B) reflecting the reduced larval olfactory system which also corresponds to the lower amount of expressed odorant receptors previously described (41 in larvae compared to 111 in adults) [94].

In a previous study regarding the stink glands of *T. castaneum* [95], *Tc*OBP8A, *Tc*OBP6B, *Tc*CSP7P and *Tc*CSP7R were identified to be transcriptionally enriched in the prothoracic glands compared to general anterior abdominal tissue. Interestingly *Tc*OBP8A and *Tc*OBP6B were also enriched in antennae and mouthparts, whereas *Tc*CSP7P was only detected in embryo and pupa. It seems that in *Tribolium* these OBPs are not only involved in reception of odorants/pheromones but also in production or release of such semiochemicals, as postulated for some lepidoptera [26, 49].

To relate our transcriptome data to protein detection in the adult antenna, we additionally performed MALDI-TOF-TOF MS with antennal extracts. We were able to identify fingerprints of 14 of the 70 candidates in antennae on protein level (Figure 1B). Thus, we found at least one tryptic fragment with an ion score above 50 that maps uniquely to the AA sequence of one of the CSPs or OBPs (Additional file 3, Table S2). We identified three highly expressed CSPs (*Tc*CSP7A, *Tc*CSP7G, *Tc*CSP7R), the three highest expressed classic OBPs (*Tc*OBP4C, *Tc*OBP4D, *Tc*OBP4E), four of the highly expressed ABPII subclass (*Tc*OBP5C, *Tc*OBP5D, *Tc*OBP5H, *Tc*OBP9B), and four C-OBPs (*Tc*OBP7E, *Tc*OBP7J, *Tc*OBP10A, *Tc*OBP10B). All identified tryptic fragments belong to genes that are transcribed in the antennae, therefore confirming their expression also on a protein level.

### Phylogenetic considerations in respect to the expression of the CSPs and OBPs

**Figure 3:**
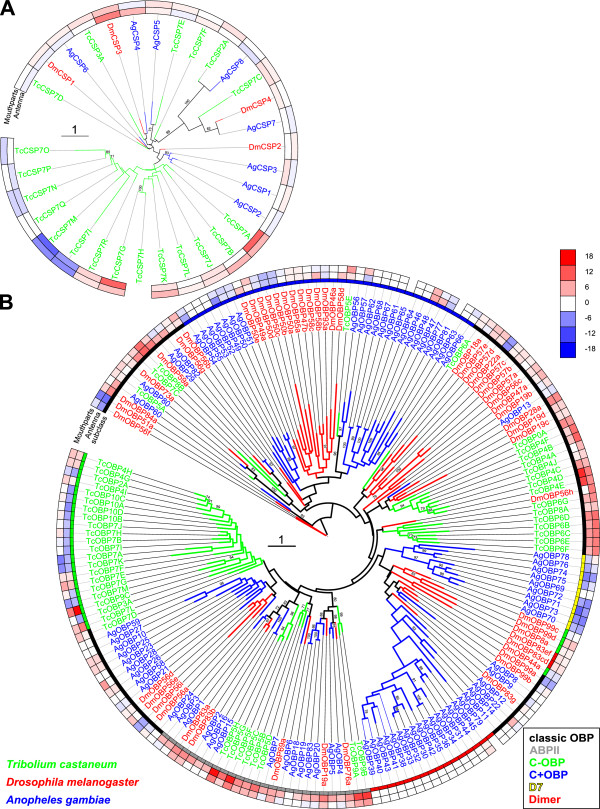
**Mid-point rooted phylogenetic tree of CSP (A) and OBP (B) sequences from** ***Tribolium castaneum*** **(green branches),** ***Drosophila melanogaster*** **(red branches), and** ***Anopheles gambiae*** **(blue branches).** Outer rings represent the expression in antennae and ‘mouthparts’ (*Tribolium*: palps, mandible, labrum and labium; *Drosophila*: palp and proboscis; *Anopheles*: maxillary palp) as log2 fold change compared to body corresponding to the scale in the right middle. The scale bars within the trees represent 1 amino acid substitution per site. Inner ring in B indicates the phylogenetic subclass (classic in black, ABPII in grey, C-OBP in green, C + OBP in blue, D7 in yellow, Dimer in red). Numbers on branches show values of 100 times replication bootstrap analysis higher than 70.

The majority of the 50 OBPs and 20 CSPS of *T. castaneum* are arranged in clusters like in other insects e.g. *D. melanogaster* [22], *An. gambiae* [96], the honey bee *Apis mellifera* [21] and silk moth *Bombyx mori* [97]. The CSPs are organized in arrays of two, six and ten genes on the seventh chromosome, only two (*Tc*CSP2A, *Tc*CSP3A) are non-clustered and located on chromosome 2 and 3. Most of the classic OBPs are arranged in two large arrays on chromosome 4 and 6, only six are interspersed (*Tc*OBP5A, *Tc*OBP6A, *Tc*OBP7C, *Tc*OBP7D, *Tc*OBP8A and *Tc*OBP8B). Six of the eight ABPIIs are located on chromosome 5 with three genes per cluster, the remaining two are close together on Chromosome 9. Nine of the 21 C-OBPs are located in a cluster on chromosome 7, close to the interspersed classic OBP *Tc*OBP7D, that is phylogenetically the closest relative classic OBP to all C-OBPs. Additional three C-OBPs form a cluster on chromosome 4, four on chromosome 10 and the remaining 5 C-OBPs are interspersed on chromosome 2, 7 and 9. The only C + OBP (*Tc*OBP5E) is located next to the second ABPII cluster on chromosome 5, but is phylogenetically unrelated to this group. All other OBPs carrying an additional cysteine are randomly distributed over the genome. The presence of clusters of phylogenetically related genes in all investigated insects can be explained by their origin from gene duplication events within the respective lineage but the fact that the clusters are conserved within different *Drosophilidae* indicates some constraints that stabilize the clusters [98]. One possible explanation for the maintaining of the clusters is the sharing of regulative elements [99], however, our expression data do not support this theory since genes from the same cluster (Figure 1B, indicated by horizontal brackets) show partially unrelated expression. Most likely more sophisticated methods are needed to understand the complex interplay of regulative elements within a cluster as recently shown for regulatory elements of odorant receptors in *Drosophila* [100] and their distribution within clusters. An interspecies comparison between *T. castaneum, D. melanogaster*, and *An. gambiae* regarding the expression level in a phylogenetic context revealed that some expression features found in *T. castaneum* are conserved between these species. The majority of the CSPs of all species are expressed in all tissues (Figure 3A). The classic OBPs in the branch holding genes of all three species are mainly enriched in antennae and/or mouthparts (Figure 3B). Only three – namely *Dm*OBP22a, *Dm*OBP56f, *Dm*OBP51a – are clearly underrepresented in the main chemosensory tissues. Also the antennal expression of *T. castaneum* ABPIIs is consistent in the other species (Figure 3B). All members of this subgroup except *AgOBP18* are enriched in antennae and the highest expressed OBPs within each species belong to this group.

**Figure 4:**
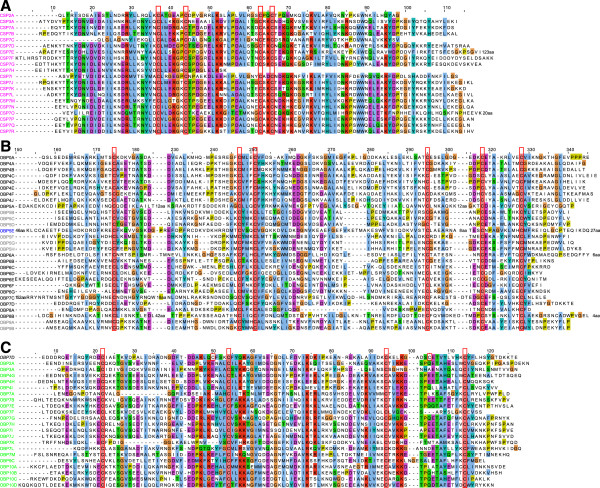
**Multiple alignments of CSPs (A), OBPs (B), and C-OBPs including TcOBP7D (C) made with ClustalW2**[94]**and visualized with Jalview 2.0.1**[95]**.** OBP8B was manually adapted to fit the conserved cysteine pattern. The N-terminal regions of OBP7C, 8B and 5E are truncated, as well as a portion between C1 and C2 of OBP5A and OBP7C, and the C-terminus of OBP5E and OBP6A (the number of deleted AA is indicated, respectively). Cysteines are indicated by red frames. CSPs are indicated in pink, classic OBPs in black, ABPIIs in grey, C-OBPs in green, and the C + OBP in blue.

The comparison of the C-OBPs of *T. castaneum*, *An. gambiae*, and *D. melanogaster* revealed that they are polyphyletic (Figure 3B) as previously shown by Vieira and Rozas [17]. The phylogenetic analysis as well as the chromosomal clustering indicates that in *T. castaneum* this large expanded group is together with the classic OBP *Tc*OBP7D most likely derived from a common ancestor. This is similar to the situation in *A. mellifera*, where a monophyletic group of C-OBPs (*Am*OBP14 to *Am*OBP21) clusters together with the classic OBP *Am*OBP13 both on the genomic localization and on the phylogenetic level [21]. However, even though the C-OBPs of different species are polyphyletic in their origin, they are in general highly and equally expressed in all tissues indicating a broad function. The loss of a disulfide bridge might increase their binding flexibility to serve different binding tasks [10, 20, 101]. The C-OBPs (21 in *T. castaneum*, four in *D. melanogaster*) actually represent similarly to the CSPs (20 in *T. castaneum*, four in *D. melanogaster*, eight in *An. gambiae*) a large expansion in *T. castaneum* and are mostly not antennae- or mouthpart-specifically expressed. Therefore, these proteins might not be mainly involved in chemosensory detection but might have additional roles such as detoxification which has been discussed for *D. sechelia* [102].

## Conclusion

Our *T. castaneum* expression analysis revealed expression of most CSPs and C-OBPs in various body parts, whereas expression of classical OBPs and ABPIIs is mainly restricted to the antennae and mouthparts. These data are consistent with previous observations in different insects [48], like *A. meliffera* [21, 65], *An. gambiae* [42], *B. mori* [103] and the large black chafer *Holotrichia parallela* [104]. Systematic OBP knockdowns in *D. melanogaster* show their necessity for correct olfactory behavioral responses and indicate a combinatorial OBP-dependent odorant recognition [38]. Our comparative expression data suggest that within the classic OBPs, especially the ABPII subgroup has a specific role in olfaction, since all members of *T. castaneum*, *An. gambiae*, and *D. melanogaster* are highly expressed and enriched in the antennae. Moreover, this group contains some of the most prominent OBPs such as *D. melanogaster* LUSH involved in pheromone detection [35, 41], *An. gambiae Ag*OBP4 that forms cooperative heteromers with other OBPs [105], and *Ag*OBP1 that mediates indole detection to find blood meals [39] and is co-expressed with other ABPIIs (*Ag*OBP3, *Ag*OBP4, *Ag*OBP19) [106].

Most OBPs of *T. castaneum* are arranged in clusters in the genome. The only exceptions are *Tc*OBP5A, *Tc*OBP6A, *Tc*OBP7C, *Tc*OBP7D, *Tc*OBP8A, and *Tc*OBP8B. Intriguingly three of them (*Tc*OBP5A, *Tc*OBP6A, *Tc*OBP7D) show an atypical, ubiquitous expression and four differ massively from the average size of 143 AA: *Tc*OBP8A 106 AA; *Tc*OBP5A 176 AA, *Tc*OBP8B 200 AA, and *Tc*OBP7C 320 AA (Figure 4). Interestingly, a bootstrap value of 100 indicates orthology of TcOBP5A with a single widely expressed OBP in *An. gambiae* (AgOBP80) and *D. melanogaster* (DmOBP73a) (Figure 3), which seem to have also single orthologues in *B. mori*, the pea aphid *Acyrthosiphon pisum*, and the body louse *Pediculus humanus* [17].

In contrast to the ABPIIs and classic OBPs, the CSPs and C-OBPs show a more broad expression indicating a more versatile function in transport of hydrophobic chemicals involved in various processes. In *T. castaneum*, several functions besides semiochemical reception are implicated: *Tc*CSP7P and *Tc*CSP7R are highly enriched in odoriferous glands [95] and may be involved in the secretion of semiochemicals or defensive products; *Tc*OBP10B and *Tc*CSP7D are up-regulated after cry toxin exposure [107] indicating a function in detoxification or the innate immune system of *T. castaneum. Tc*OBP7F is transferred during copulation via seminal fluids [108] similar to the yellow fever mosquito *Aedes aegyti* [109] to potentially mark fertilized eggs, as also described for *Helicoverpa* moths [50]. Many more functions of CSPs in insects have sporadically been described, such as involvement in limb regeneration in the American cockroach *Periplaneta americana* [53], presence in the female reproductive organs of the migratory locust *Locusta migratoria* [110], involvement in embryonic integument formation in *A. mellifera* [51], response to an insecticide in the silverleaf whitefly *Bemisia tabaci* [111] and *B. mori* [112] *,* detergent like function in the proboscis of two *Helicoverpa* species [113]. Taken together, some CSPs seem to participate in chemoreception, however, most of them might have more general functions involved in the release of semiochemicals [26], development [51], reproduction [50, 108, 109, 114], food intake [113], and in the drug/immune response [52, 107, 111, 112].

## Availability of supporting data

The complete transcriptomics dataset including all relevant parameters has been deposited to the National Center for Biotechnology Information (NCBI) database repository ‘Gene Expression Omnibus’ (GEO accession number: GSE63162) [115].

## Electronic supplementary material

Additional file 1: Table S1: New names (column 1; uploaded at iBeetle genome browser [48]) based on chromosomal localization and corresponding previous names from Richards et al. 2008 [7] (column 2; uploaded at Beetle Base) as well as Foret and Maleska [16, 63] respectively from Vieira and Rozas [12] (column 3). The AA sequence including the signal peptide with the tryptic fragments identified by MALDI-TOF MS highlighted in red (column 4). The length of the pre-peptide (column 5) and mature peptide (column 6) in AA. The predicted length of the signal peptide (column 7; based on SignalP4.1 [50]). The molecular mass of the pre-peptide (column 8) and the mature peptide (column 9). The superfamily identified by the conserved domain database [51] (column 10), as well the probability as e-value (column 11). The confidence of the signal peptide [50] (column 12). The number of cysteines in the mature peptide (column 13). The Cysteine formula adjusted to the six highly conserved cysteines (column 14; including C-OBPs). The isoelectric point (based on endmemo [116], column 15). The position of alpha helices (based on jpred [117], column 16). Whether it was confirmed by cloning from cDNA (column 17). Primer sequences used for cloning from cDNA (columns 18 and 19). The reads per kilobase of exon model per million mapped reads of the different tissue samples (columns 20–34) The calculated fold-changes over body, and the corresponding p-values (DESeq package [80], column 35–42). (XLSX 74 KB)

Additional file 2: Figure S1: Midpoint-rooted phylogenetic tree of CSP (A) and OBP (B) sequences from *Tribolium castaneum* (green branches), *Drosophila melanogaster* (red branches), and *Anopheles gambiae* (blue branches). Outer rings represent the expression in body, ‘mouthparts’ (*Tribolium*: palps, mandible, labrum and labium; *Drosophila*:palp and proboscis; *Anopheles*: maxillary palp) and antenna as percentage compared to the highest expressed gene according to the scale in the right middle. Please note that the methods used to obtain the different expression data (RNAseq and microarray) are not directly comparable. Thus, this figure can only give an impression of the tissue-specific abundance of the transcripts. The scale bars within the trees represent 1 amino acid substitution per site. Inner ring in B indicates the phylogenetic subclass (classic in black, ABPII in grey, C-OBP in green, C + OBP in blue, D7 in yellow, Dimer in red). Numbers on branches show values of 100 times replication bootstrap analysis higher than 70. (PDF 504 KB)

Additional file 3: Table S2: Proteins of antennal extracts identified by MALDI-TOF-TOF MS-fingerprinting: accession number, molecular weight, protein isoelectric point, peptide count, ion score, and Pfam prediction. (XLSX 24 KB)

Additional file 4: Figure S2: Comparison of expression level of CSPs and OBPs in male and female heads (missing antennae but including mouthparts). Scatter plot of the RPKM values of the CSPs (in pink) and OBPs (classic in black, ABPII in grey, C-OBP in green, C + OBP in blue). The dotted lines represent a fivefold difference. (PDF 88 KB)

Additional file 5: Figure S3: Comparison of expression level of CSPs and OBPs in male and female mouthparts. Scatter plot of the RPKM values of the CSPs (in pink) and OBPs (classic in black, ABPII in grey, C-OBP in green, C + OBP in blue). The dotted lines represent a fivefold difference. (PDF 87 KB)

Additional file 6: Figure S4: Comparison of expression level of CSPs and OBPs in male and female legs. Scatter plot of the RPKM values of the CSPs (in pink) and OBPs (classic in black, ABPII in grey, C-OBP in green, C + OBP in blue). The dotted lines represent a fivefold difference. (PDF 84 KB)

Additional file 7: Figure S5: Comparison of expression level of CSPs and OBPs in male and female bodies. Scatter plot of the RPKM values of the CSPs (in pink) and OBPs (classic in black, ABPII in grey, C-OBP in green, C + OBP in blue). The dotted lines represent a five fold difference. (PDF 84 KB)
