# Supplementary figures and images for: Tissue-specific transcriptomics, chromosomal localization, and phylogeny of chemosensory and odorant binding proteins from the red flour beetle Tribolium castaneum reveal subgroup specificities for olfaction or more general functions
Source: BMC Genomics. 2014 Dec 18;15:1141. doi: 10.1186/1471-2164-15-1141 (PMC4377858; doi:10.1186/1471-2164-15-1141)

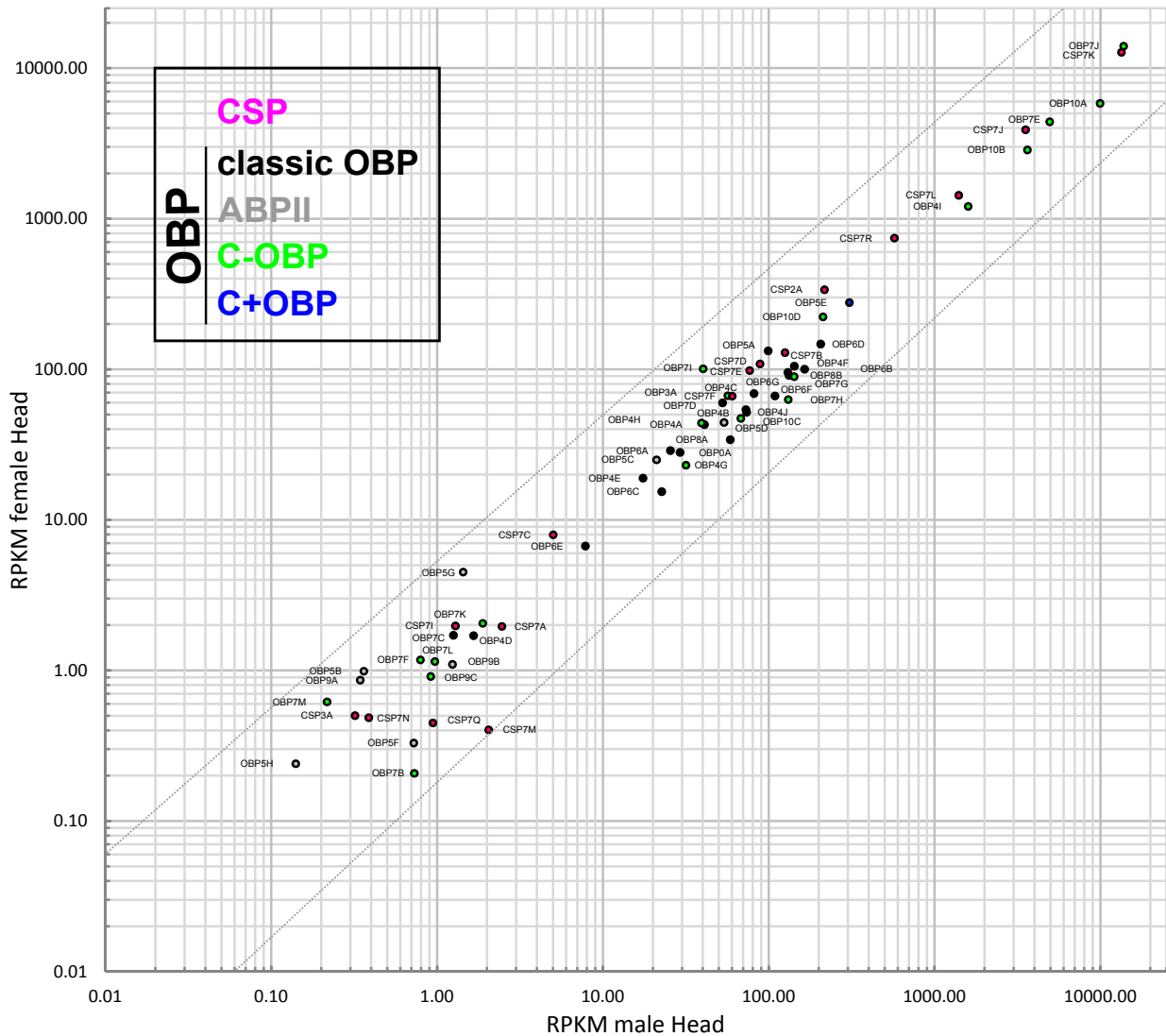

Supplement: Supplementary file 4 — Additional file 4: Figure S2: Comparison of expression level of CSPs and OBPs in male and female heads (missing antennae but including mouthparts). Scatter plot of the RPKM values of the CSPs (in pink) and OBPs (classic in black, ABPII in grey, C-OBP in green, C + OBP in blue). The dotted lines represent a fivefold difference. (PDF 88 KB) [file 12864_2014_6882_MOESM4_ESM.pdf]

RPKM female Mouthparts

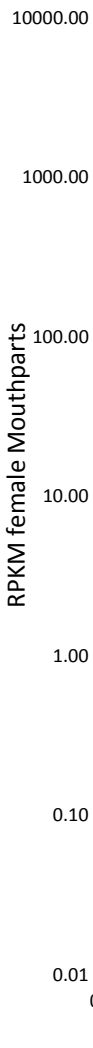

RPKM male Mouthparts

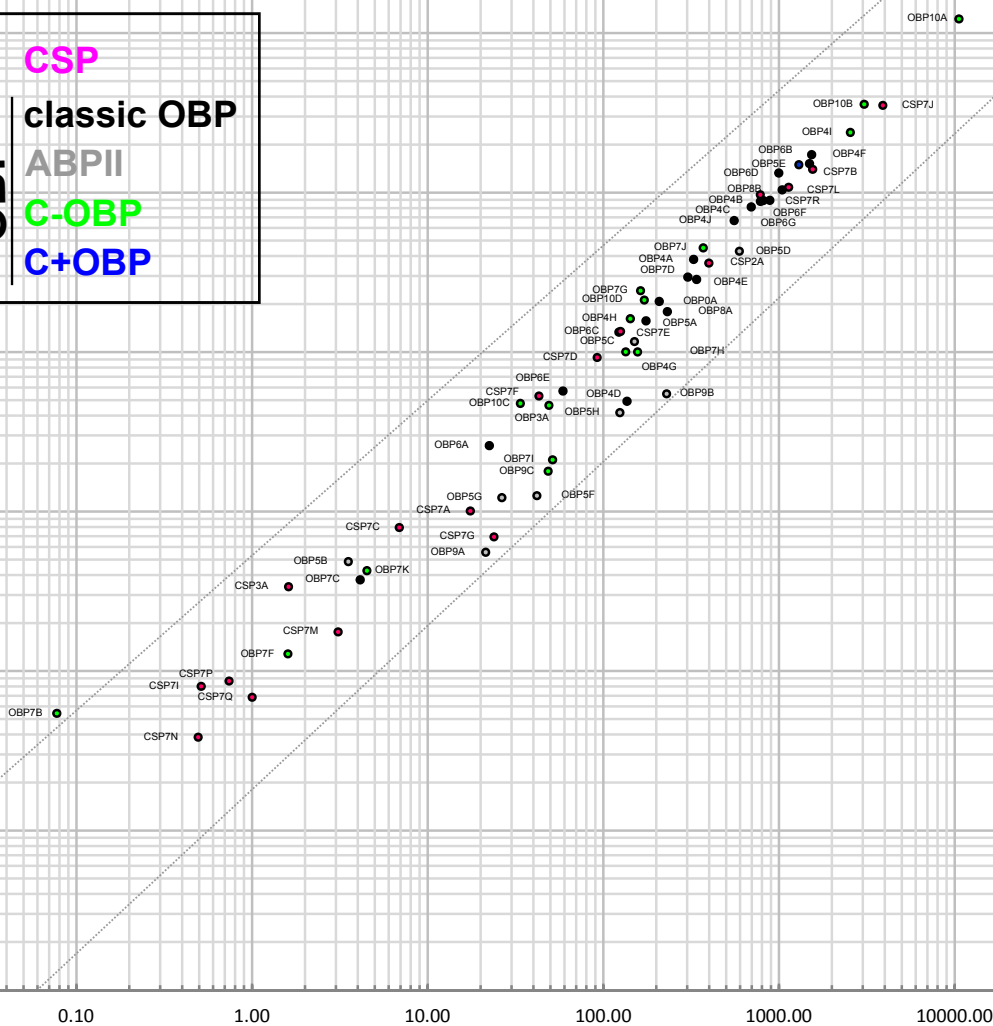

Supplement: Supplementary file 5 — Additional file 5: Figure S3: Comparison of expression level of CSPs and OBPs in male and female mouthparts. Scatter plot of the RPKM values of the CSPs (in pink) and OBPs (classic in black, ABPII in grey, C-OBP in green, C + OBP in blue). The dotted lines represent a fivefold difference. (PDF 87 KB) [file 12864_2014_6882_MOESM5_ESM.pdf]

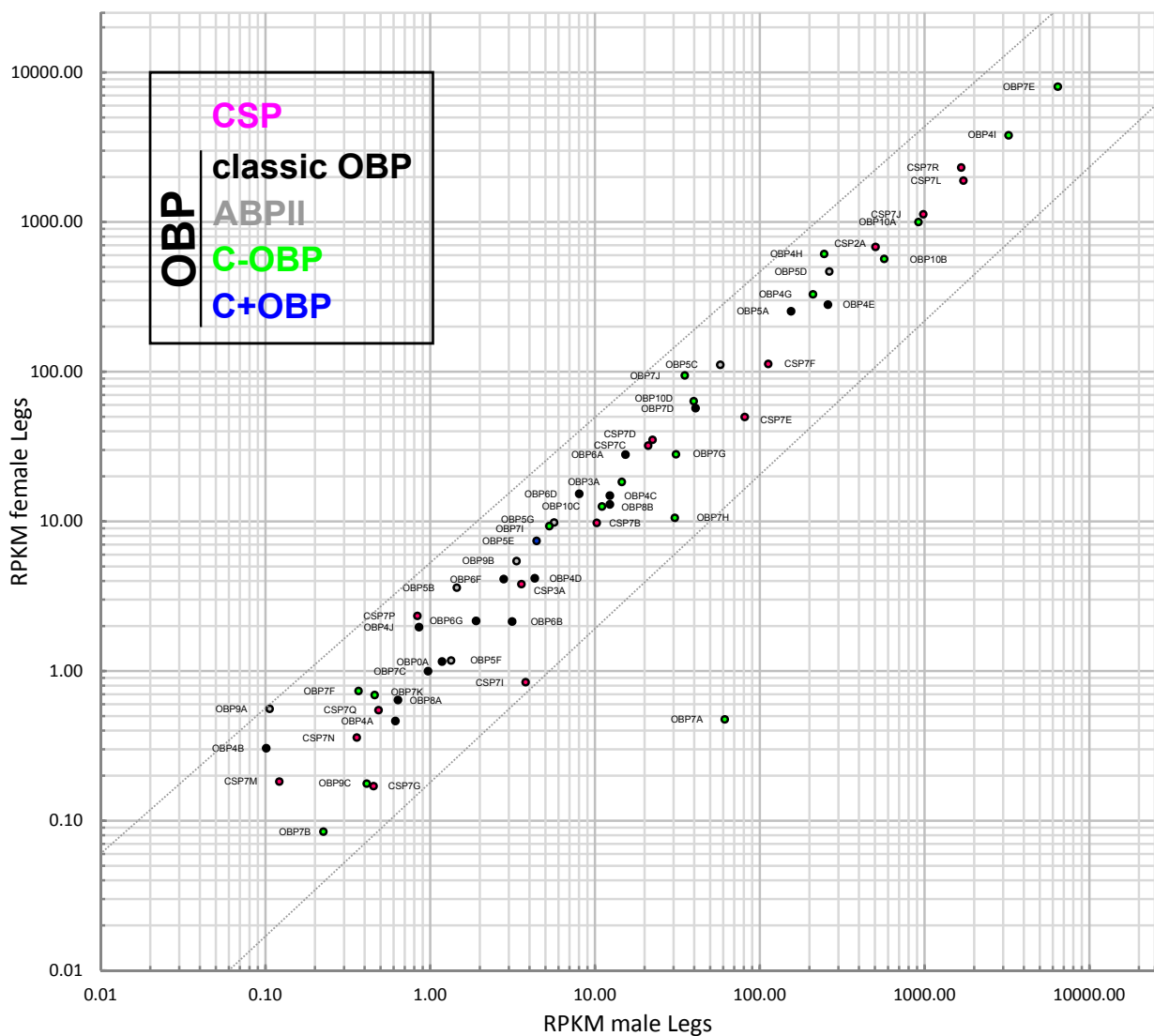

Supplement: Supplementary file 6 — Additional file 6: Figure S4: Comparison of expression level of CSPs and OBPs in male and female legs. Scatter plot of the RPKM values of the CSPs (in pink) and OBPs (classic in black, ABPII in grey, C-OBP in green, C + OBP in blue). The dotted lines represent a fivefold difference. (PDF 84 KB) [file 12864_2014_6882_MOESM6_ESM.pdf]

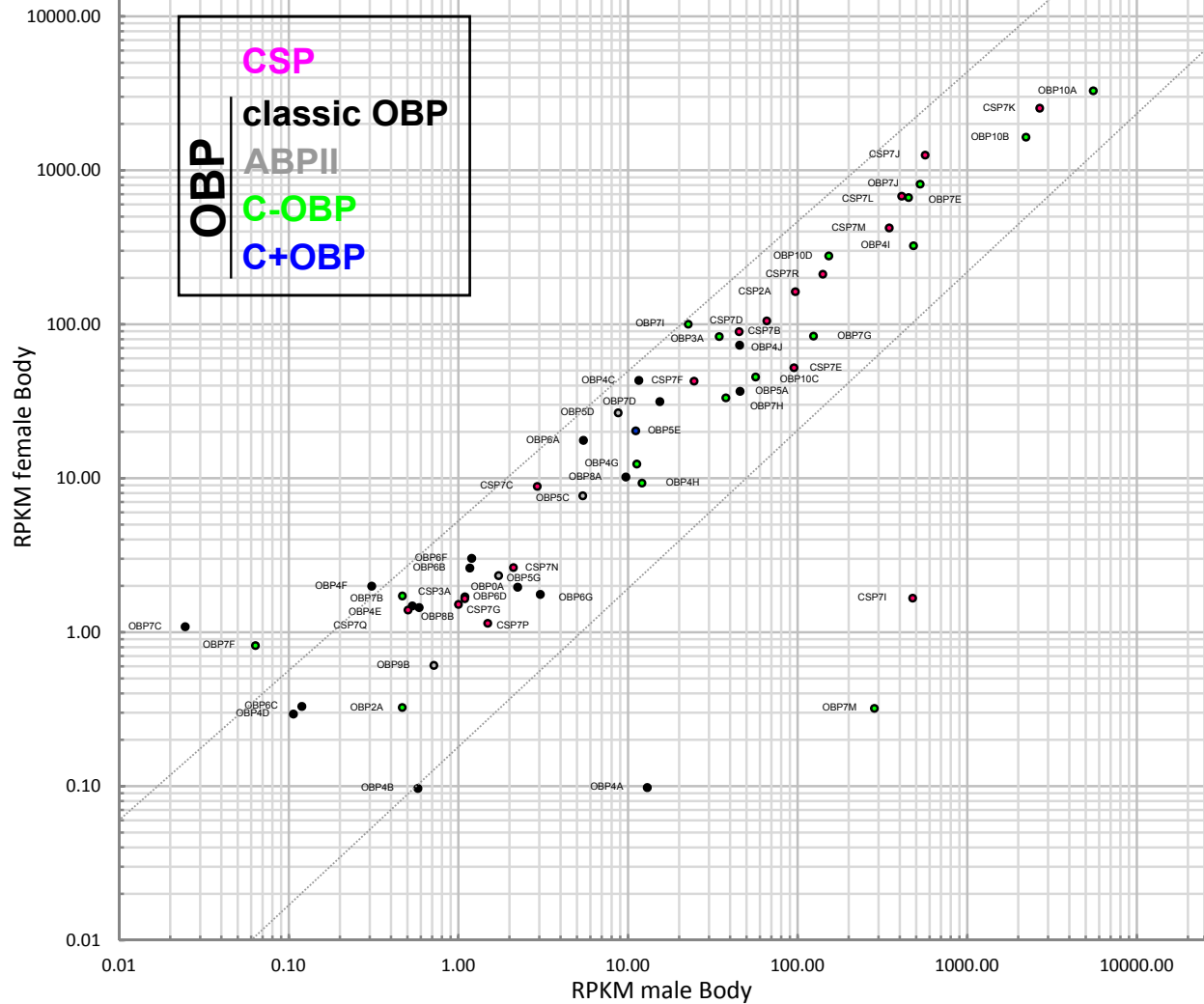

Supplement: Supplementary file 7 — Additional file 7: Figure S5: Comparison of expression level of CSPs and OBPs in male and female bodies. Scatter plot of the RPKM values of the CSPs (in pink) and OBPs (classic in black, ABPII in grey, C-OBP in green, C + OBP in blue). The dotted lines represent a five fold difference. (PDF 84 KB) [file 12864_2014_6882_MOESM7_ESM.pdf]
