# Supplementary material for: Tissue-specific transcriptomics, chromosomal localization, and phylogeny of chemosensory and odorant binding proteins from the red flour beetle Tribolium castaneum reveal subgroup specificities for olfaction or more general functions
Source: BMC Genomics. 2014 Dec 18;15:1141. doi: 10.1186/1471-2164-15-1141 (PMC4377858; doi:10.1186/1471-2164-15-1141)
Supplement: Supplementary file 2 — Additional file 2: Figure S1: Midpoint-rooted phylogenetic tree of CSP (A) and OBP (B) sequences from Tribolium castaneum (green branches), Drosophila melanogaster (red branches), and Anopheles gambiae (blue branches). Outer rings represent the expression in body, ‘mouthparts’ (Tribolium: palps, mandible, labrum and labium; Drosophila:palp and proboscis; Anopheles: maxillary palp) and antenna as percentage compared to the highest expressed gene according to the scale in the right middle. Please note that the methods used to obtain the different expression data (RNAseq and microarray) are not directly comparable. Thus, this figure can only give an impression of the tissue-specific abundance of the transcripts. The scale bars within the trees represent 1 amino acid substitution per site. Inner ring in B indicates the phylogenetic subclass (classic in black, ABPII in grey, C-OBP in green, C + OBP in blue, D7 in yellow, Dimer in red). Numbers on branches show values of 100 times replication bootstrap analysis higher than 70. (PDF 504 KB) [file 12864_2014_6882_MOESM2_ESM.pdf]

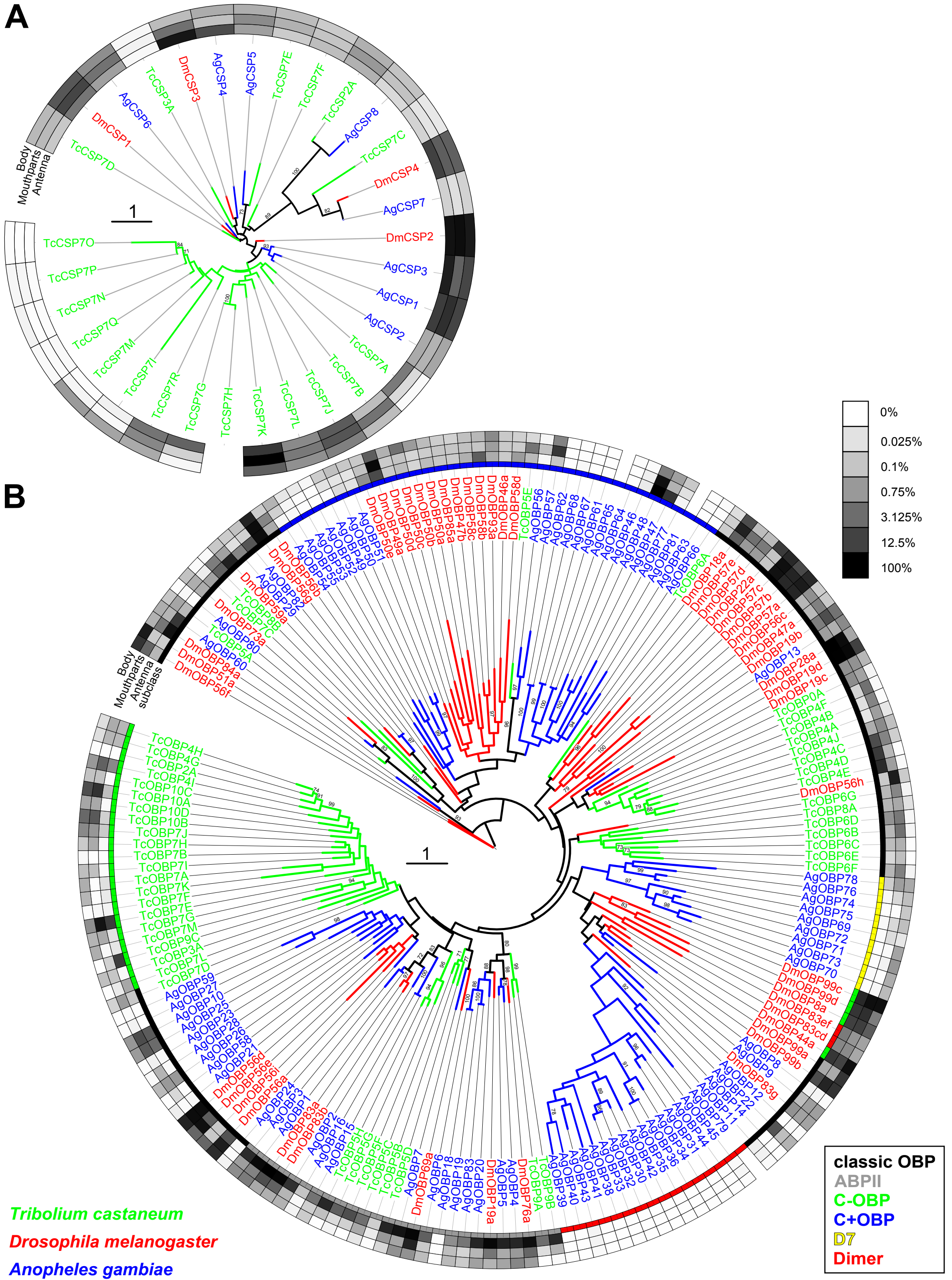

A
Body
Mouthparts
Antenna
1
TcCSP7D
DmCSP1
AgCSP6
TcCSP3A
DmCSP3
AgCSP4
AgCSP5
TcCSP7E
TcCSP7F
TcCSP2A
AgCSP8
TcCSP7C
DmCSP4
AgCSP7
DmCSP2
AgCSP3
AgCSP1
AgCSP2
TcCSP7A
TcCSP7B
TcCSP7J
TcCSP7L
TcCSP7K
TcCSP7H
TcCSP7G
TcCSP7R
TcCSP7I
TcCSP7M
TcCSP7Q
TcCSP7N
TcCSP7P
TcCSP7O
0%
0.025%
0.1%
0.75%
3.125%
12.5%
100%
B
Body
Mouthparts
Antenna
subclass
1
DmOBP56f
DmOBP51a
DmOBP84a
AgOBP60
TcOBP5A
AgOBP80
DmOBP73a
TcOBP7C
TcOBP8B
DmOBP59a
AgOBP29
AgOBP82
DmOBP56g
DmOBP56b
AgOBP54
AgOBP55
AgOBP53
AgOBP52
AgOBP49
AgOBP51
AgOBP50
DmOBP49a
DmOBP50e
DmOBP50d
DmOBP50c
DmOBP50b
DmOBP50a
DmOBP85a
DmOBP47b
DmOBP58c
DmOBP58b
DmOBP93a
DmOBP46a
DmOBP58d
TcOBP5E
AgOBP56
AgOBP57
AgOBP62
AgOBP68
AgOBP67
AgOBP61
AgOBP65
AgOBP64
AgOBP46
AgOBP48
AgOBP47
AgOBP77
AgOBP81
AgOBP63
AgOBP66
TcOBP6A
DmOBP18a
DmOBP57e
DmOBP57d
DmOBP22a
DmOBP57c
DmOBP57b
DmOBP57a
DmOBP56c
DmOBP47a
DmOBP19b
AgOBP13
DmOBP28a
DmOBP19d
DmOBP19c
TcOBP0A
TcOBP4F
TcOBP4B
TcOBP4A
TcOBP4J
TcOBP4C
TcOBP4D
TcOBP4E
DmOBP56h
TcOBP6G
TcOBP8A
TcOBP6D
TcOBP6B
TcOBP6C
TcOBP6E
TcOBP6F
AgOBP78
AgOBP76
AgOBP74
AgOBP75
AgOBP69
AgOBP72
AgOBP71
AgOBP73
AgOBP70
DmOBP99c
DmOBP99d
DmOBP8a
DmOBP83ef
DmOBP83cd
DmOBP44a
DmOBP99a
DmOBP99b
AgOBP8
AgOBP9
DmOBP83g
AgOBP12
AgOBP22
AgOBP14
AgOBP11
AgOBP79
AgOBP45
AgOBP44
AgOBP31
AgOBP34
AgOBP36
AgOBP35
AgOBP42
AgOBP30
AgOBP32
AgOBP33
AgOBP41
AgOBP43
AgOBP38
AgOBP40
AgOBP39
TcOBP9B
TcOBP9A
DmOBP76a
AgOBP4
AgOBP5
DmOBP19a
AgOBP20
AgOBP83
AgOBP19
AgOBP18
AgOBP6
DmOBP69a
AgOBP7
TcOBP5D
TcOBP5B
TcOBP5C
TcOBP5F
TcOBP5G
TcOBP5H
AgOBP15
AgOBP16
AgOBP2
DmOBP83b
DmOBP83a
AgOBP1
AgOBP3
AgOBP24
DmOBP56a
DmOBP56i
DmOBP56e
DmOBP56d
AgOBP21
AgOBP58
AgOBP26
AgOBP28
AgOBP23
AgOBP25
AgOBP10
AgOBP27
AgOBP59
TcOBP7D
TcOBP7L
TcOBP3A
TcOBP9C
TcOBP7M
TcOBP7G
TcOBP7E
TcOBP7F
TcOBP7K
TcOBP7A
TcOBP7I
TcOBP7B
TcOBP7H
TcOBP7J
TcOBP10B
TcOBP10D
TcOBP10A
TcOBP10C
TcOBP4I
TcOBP2A
TcOBP4G
TcOBP4H
Tribolium castaneum
Drosophila melanogaster
Anopheles gambiae
classic OBP
ABPII
C-OBP
C+OBP
D7
Dimer
